# Association between affective temperaments and psychosomatic symptoms in women with Hashimoto’s thyroiditis

**DOI:** 10.1371/journal.pone.0290066

**Published:** 2023-08-15

**Authors:** Gordana Stanić, Snežana Marinković, Jelena Milin Lazović, Dragana Ignjatović Ristić

**Affiliations:** 1 Academy of Applied Studies Belgrade Department of School of Applied Health Sciences, Belgrade, Serbia; 2 Department of Psychiatry, Facult of Medical Sciences, University of Kragujevac, Kragujevac, Serbia; 3 Special Hospital for Thyroid Gland and Metabolism Disease Zlatibor, Zlatibor, Serbia; 4 Faculty of Medicine, University of Belgrade, Belgrade, Serbia; Virginia Tech Carilion School of Medicine, UNITED STATES

## Abstract

**Background:**

Hashimoto’s thyroiditis (HT) is a prevalent autoimmune disease of thyroid gland with a shared immunological mechanism with mood disorders. Affective temperament (AT) is a biologically determined personality trait that has been linked to mood disorders. The aim of this study was to examine the association between dominant AT and levels of psychosomatic symptoms in women newly diagnosed with HT in comparison to clinically healthy subjects.

**Methods:**

The observational cross-sectional study with nested case control study was involving 146 consecutive participants, who were divided into three groups. The two study groups consisted of women with HT (73), including 49 with hypothyroid HT and 24 with euthyroid HT, and the third group was a control group of healthy participants (73). The Serbian version of the TEMPS-A was utilized to assess AT, while the 4DSQ was used to measure psychosomatic symptoms.

**Results:**

The results showed that hyperthymic AT was dominant in all examined groups. The groups with HT differed from the control group in terms of depressive and cyclothymic AT. Furthermore, the study found higher levels of psychosomatic symptoms in the group with HT compared to the control group, with significant differences in distress (p = 0.005) and somatization (p = 0.023) levels. All AT was associated with levels of psychosomatic symptoms in subjects with hypothyroid HT. In contrast, in subjects with euthyroid HT, the association was only found between depressive and cyclothymic AT with distress and depression levels, as well as between somatization and cyclothymic AT. No association was found between AT and anxiety levels in subjects with euthyroid HT.

**Conclusion:**

The research found differences between study groups in the association between AT and levels of psychosomatic symptoms. Further research with a larger sample size is necessary to more clearly define the associations between affective temperaments and psychosomatic symptoms in women with euthyroid and hypothyroid HT.

## Introduction

Hashimoto’s thyroiditis (HT) is a prevalent autoimmune disease that is characterized by chronic autoimmune thyroiditis, which can be accompanied by thyroid dysfunction. In certain number of patients with HT we encounter a euthyroid function of the thyroid gland [1–3], but with the most of the patients the hypothyroidism is developed [4]. The incidence of HT is estimated to be 5% in the general population, with a higher prevalence among women [1–3, 5, 6].

It is known that there is a common immune pathogenesis for autoimmune disorders of the thyroid gland and mood disorders [7]. Several previous studies suggest physical and mental health impairments in patients with HT [8]. The findings of previous research indicate that there is an association between HT and mood disorders, but also a high risk of the psychosomatic symptoms development [3, 9, 10]. Epidemiological studies indicate an association between depression and anxiety in people suffering from HT, as well as the risk of psychiatric illnesses [11]. Stress affects the endocrine and immune mechanisms involved in HT, hyperactivity of the hypothalamic-pituitary-adrenal stress axis results in increased cortisol levels in the bloodstream, causing decreased regulation of thyroid hormones and hypothyroidism [12]. Long-term stress is a big risk for the development of the disease, so it is crucial to take into account the mental state of HT and new stress at the beginning of the disease. Stress can be associated with depression, anxiety and perception of somatic symptoms [13]. Somatization implies physical symptoms that cannot be explained by physiological processes of the illness, which are associated with the changed autoimmune balance and emotional conditions, anxiety and depression [14, 15].

Affective temperament (AT) is biologically determined and reflects time-stable personality traits and emotional reactions [16]. It is believed that AT, relatively stable expressions of affect, are at the basis of the development and expression of mood disorders [16, 17]. AT can be described as sensitivity to mood disorders, anxiety and depression [16–19]. Temperament traits are associated with serotonergic activity and are studied in many psychosomatic disorders [20–22]. Several studies have investigated the association between AT and autoimmune diseases [22]. In literature, we encounter data pointing out that the presence of certain AT affects the expression of psychiatric comorbidity with patients suffering from autoimmune diseases [21, 22]. More recent studies have established correlations between a greater numbers of AT that are responsible for mood disorders and the frequency of occurrence of depression and anxiety [22]. However, there is a scarcity of data on the relationship between dominant affective temperaments and levels of psychosomatic symptoms in women with newly diagnosed HT. There is a need to pay more attention to mental health and take into account psychosomatic symptoms in the growing number of women suffering from HT [9], because this illness affects the disorder of emotions in sufferers [5–7]. Our hypothesis is that determining of the dominant AT could provide useful information about the sensitivity to psychosomatic symptoms in these patients.

**The aim of the study** was to examine the association between dominant affective temperaments and levels of psychosomatic symptoms (distress, depression, anxiety and somatization) in women newly diagnosed with Hashimoto’s thyroiditis in comparison to clinically healthy subjects.

## Materials and methods

### Design of study

The study was designed as observational cross-sectional study with nested case control study and was carried out from August 2021 to February 2022 at the Special hospital for thyroid gland and metabolism disease Zlatibor, Serbia. The study was carried out in accordance with the ethical principles of the Helsinki Declaration. All respondents gave their informed consent to participate in the study, which was approved by Ethics Committee (code EC–01–9344).

### Participants

The research involved in total 146 consecutive female subjects, who were between 18 and 66 years old. Subjects were divided into three groups. Two examined groups consisted of women with newly diagnosed HT (73), of which one group consisted of subjects with hypothyroid HT (49) and the other group of subjects with euthyroid HT (24). Group homogenization was performed based on the diagnosis of hypothyroid or euthyroid HT. The third control group consisted of clinically healthy subjects (73), who were confirmed not to have thyroid disease or HT. They were matched by gender and age.

*The inclusion criteria for the study group with a diagnosis of HT were*: female patients, with diagnosis of HT, hypothyroidism and euthyroid thyroid function, who were over the age of 18, and who had not been previously treated for thyroid diseases and gave their voluntary consent to participate in the study. *The inclusion criteria for the control*, *healthy group* were: female subjects from the general population, healthy volunteers, who do not have thyroid disease or HT diagnosed, without somatic and psychiatric diseases, they are healthy, have no clinical indicators and do not provide data on thyroid disease or psychiatric diseases in the anamnesis. Female subjects with HT and control groups were matched by age and gender. *Exclusion criteria for all subjects (newly diagnosed HT and control*, *healthy subjects) were*: pregnancy, presence of other comorbidities (other autoimmune and endocrine diseases; malignancies; rheumatoid; renal; psychiatric disorders of cognition that interfere with filling out questionnaires, diagnosis or treatment of psychosis, depression, anxiety, bipolar disorder and neurological diseases, dementia or neuronal damage whose treatment requires the use of corticosteroids), then subjects who had been taking drugs that may affect the test parameters glucocorticoids, estrogens (menopausal women), thyrotrophic drugs (dopamine, amiodarone and lithium) at least three months before the study. During the research period of one year, we found 116 female subjects diagnosed with HT. During the research period, HT was detected in three male subjects, which is a small number compared to female subjects, and they were excluded from the analysis. As we had clear criteria for inclusion in the study, some subjects were excluded due to the presence of pregnancy in 9 subjects, psoriasis in four subjects, three subjects diagnosed with vitiligo, rheumatoid arthritis in three subjects, 12 subjects with diabetes mellitus type 2, previously treated malignant diseases in two subjects, psychiatric diagnosis and treatment of anxiety and depression six subjects, one subject in menopause taking estrogens in therapy, two subjects diagnosed with dementia. The control group of subjects had the same criteria for exclusion from the study, so subjects with clinically diagnosed HT and other diseases of the thyroid gland were not included, as well as subjects who gave information about all the other diseases listed in the anamnesis.

### Research protocol

The study included all consecutive outpatients female subjects, wich came for examination at the Special hospital for thyroid gland and metabolism disease Zlatibor, Serbia. The subjects underwent laboratory analyses, were clinically examined by an endocrinologist who diagnosed HT in accordance with the International Classification of Diseases E06.3 diagnostic criteria for diseases of the endocrine system and who had ultrasound findings characteristic of HT. Afterwards, the subjects were divided into three groups. The first group was composed of clinical, manifested hypothyroid form of HT, which is characterized by elevated level of thyroid stimulating hormone (TSH>4.2 mIU/L) in the blood serum, and decreased production of free serum thyroxine (FT4<10.2 nmol/L) with increased presence of TPO antibodies (TPO>34 mlU/L). The second group was composed of subjects with euthyroid HT, which is characterized by normal levels of TSH and FT4 ​​(TSH = 0.4–4.2 mIU/L (CLIA-C); FT4 = 10.2–24.5 nmol/L (CMIA) with increased level of TPO antibodies (TPO>34 mlU/L). The third group included subjects who do not have a thyroid function disorder, whose laboratory data show the reference values of TSH and FT4 for normal thyroid function of antibodies. This contol group of healthy women was selected for the study group of the included subjects with HT in accordance with the sex and age and that they do not have any of the exclusionary criteria.

Subjects who met the inclusion criteria gave their voluntary consent to participate in the study, filled out the questionnaires and had anthropometric measurements.

### Instruments

After being classified into groups, all respondents filled out sociodemographic questionnaire, the Serbian version of the TEPMS-A evaluate affective temperaments and 4DSQ for psychosomatic symptoms.

We evaluated demographic factors using a sociodemographic questionnaire, compiled for this study. This questionnaire took into consideration the age, education level, marital and work status, laboratory parameters including TSH, FT4 and TPO antibodies, and anthropometric measurements including waist circumference, body weight, body height, to calculate Body Mass Index (BMI) as the weight to height ratio (kg/m^2^).

**Temperament Evaluation of Memphis, Paris and San Diego Auto-questionnaire (TEMPS-A)** self-assessment scale shows the most important traits of affective temperaments. In this study, we used the Serbian version of the TEPMS-A questionnaire with 41 items, which exhibited construct validity and good overall internal consistency (Combah ά = 0.83), indicating stable reliability over time [23]. This scale indicates six types of temperament: depressive (1–7), cyclothymic (8–14), hyperthymic (15–21), irritable (22–29), anxiety-cognitive (30–35) and anxiety-somatic (36–41). Respondents mark true/false on the questionnaire, depending on whether the statement describes the way they usually behave or feel, whereby a correct answer is scored with one point, and an incorrect answer with 0 points. The total number of marked correct statements in a certain category is added up and the sum is divided by the total number of items in that category. Dominant affective temperaments were calculated by adding the scores of individual items [16, 23].

**The Four-Dimensional Symptom Questionnaire (4DSQ)** was used to determine the presence and levels of distress, depression, anxiety and somatization. It is a four-dimensional symptom questionnaire containing 50 claims arranged in four scales (distress, depression, anxiety and somatization) that are scored separately [24]. The reference time period in the scale is within the previous seven days. On the five-point scale, the corresponding statements are marked with:“no“, “sometimes“,“regularly“, “often“, “very often”or “constantly“. To get the scale results, the answers were evaluated with 0 for “no”, 1 for “sometimes” and 2 for other answer categories. Adding all items gives the results of the scale. The distress subscale contains 16 items and has a score range of 0–32 and the categorization of results is: mild (0–9), moderate elevated (10–20) and strongly elevated (21 and more). The depression subscale contains 6 items and has a range of 0–12, the categorization of results is: mild (0–1), moderate elevated (2–5) and strongly elevated (6 and more). The anxiety subscale contains 12 items and has a range of 0–24, the categorization of results is: mild (0–7), moderate elevated (8–12) and strongly elevated (13 and more) and somatization subscale contains 16 items and has a range of 0–32, the results can be: mild (0–10), moderate elevated (11–20) and strongly elevated (21–32).

### Statistical analysis

The collected data were verified by the authors, coded and entered into database (S1 File). Results are presented as Baseline demographic and clinical features of study population are presented as mean±standard deviation, median (25th-75th) and number (%). Graphical and matematical methods are used to examine data distribution. Groups were compared using Chi Square test. Baseline demografic, clinical features and questionare scores are analyzed with Student T test for independent samples or Mann Whitney test when two groups are compared. Data were analysed with One Way ANOVA Kruscall Wallis test when three groups are compared. For One way ANOVA significant differences Tuckey post hoc analysis was used. After Kruscall Wallis test MannWhiteney test was used as posthoc test. Spearman correlation coeffcient was used to examine correlation between 4DSQ and TEMPS-A questionare. All p value of less than 0.05 were considered statistically significant. In order to avoid the risk of random significant values due to the multiple comparisons, Bonferroni correction was applied in the analysis of all the parameters. The Bonferroni-adjusted p value needed for significance was 0.05/30, or p<0.001 was considered statistically significant, as a stricter significance threshold. To assess internal consistency within the TEMPS-A and the 4DSQ questionnaire, Chronbach’s alpha was used. Crombach alpha for the TEMPS-A questionnaire is 0.784 and for the 4DSQ 0.941, which indicates a good internal consistency of the examined questionnaires. Statistical analysis was performed by IBM SPSS Statistics 26 (IBM SPSS Inc., Chicago, IL).

## Results

Our study included 146 female respondents, average age 38.7± 7.1 the to 43.8± 11.1. By comparing the groups, levels of TSH, FT4 and Anti TPO were significantly different in all groups (p<0.05). Post hoc analysis revealed that difference was significant between hypothiroid and control group (p<0.001; p<0.001), hypothiroid and euthyroid group (p<0.001; p = 0.05) and hypothyroid and euthyroid group (p = 0.029, p<0.001) FT4 levels were significantly different between three groups. Post hoc analysis revealed difference was significant between hypothiroid and control group (p<0.001) and also hypothiroid and euthyroid group (p<0.001). There was no statistically significant difference regarding other parameters. That means the study population was homogenous (Table 1).

**Table 1:** Demographic and clinical data of study population.

Variable	Hypothiroid HT* n = 49	Euthyroid HT n = 24	Control group n = 73	Statistical significance p*
**Age, mean±sd**	43.8±11.1	38.7±7.1	42.1±10.2	0.125
**Education level, Primary/Secondary**	31 (63.3)	12 (50)	40 (54.8)	0.495
**Education level, High/ Master**	18 (36.7)	12 (50)	33 (45.2)	
**Marital status, maried, living with partner, n (%)**	17 (34.7)	6 (25)	20 (27.4)	0.338
**Marital status, single, devorced, widow, n (%)**	32 (65.3)	18 (75)	53 (72.6)	
**Work status, employed, n (%)**	37 (75.5)	18 (75)	59 (80.8)	0.760
**Work status, not epmployed, n (%)**	10 (20.4)	6 (25)	12 (16.4)	
**Work status, retired, n (%)**	2 (4.1)	0 (0)	2 (2.7)	
**TSH, Med (25th-75th)**	6.45 (5.25–10.6)	3.2 (1.89–3.95)	2.27 (1.83–3)	**<0.001**
**FT4, mean±sd**	9.09 ± 1.16	14.3 ± 2.3	12.6±3.0	**<0.001**
**Anti TPO, Med (25th-75th)**	358 (138.6–795)	194.5 (100.6–276)	17 (10–27)	**<0.001**
**BMI (kg/m** ^**2**^ )*, mean±sd	26 ± 4.2	25.1 ± 4.8	25.2±4.7	0.601
**Waist circumference (cm), mean±sd**	80.1 ± 12.5	78.6 ± 12.2	79.5±12.7	0.892

HT- Hashimoto thyroiditis; *BMI = body mass index; *Statistical analysis done with Chi squared test, One-way ANOVA and Kruscal Wallis test, Tukey and Mann Whitney test were used as poshoc tests for multiple comparation

Dominant affective temperament both in Hashimoto groups and control, healthy group was hypertymic (Table 2) The assessment of affective temperament by TEMPS-A in all groups revealed higher scores of depressive and cyclothymic temperament (p = 0.016, p<0.001) in Hashimoto group, while higher scores of hyperthymic temperament were present in control group (p = 0.019).

**Table 2:** Affective temperament by TEMPS-A in study population.

Affective temperament TEMPS A	Hypothiroid HT group		Euthyroid HT group		Control group		Statistical significance P*
	mean±sd	Med (25th-75th percentile)	mean±sd	Med (25th-75th percentile)	mean±sd	Med (25th-75th percentile)	
**Depresive**	0.11 ± 0.18	0 (0–0.14)	0.14 ± 0.19	0.07 (0–0.14)	0.07 ± 0.14	0 (0–0)	**0.042**
**Ciclotymic**	**0.46 ± 0.34**	**0.43 (0.14–0.71)**	**0.49 ± 0.34**	**0.43 (0.14–0.86)**	0.23 ± 0.3	0.07 (0–0.43)	**<0.001**
**Hypertymic**	**0.65 ± 0.24**	**0.71 (0.43–0.86)**	**0.7 ± 0.22**	**0.71 (0.57–0.86)**	**0.74 ± 0.25**	**0.86 (0.57–0.86)**	0.051
**Irritabile**	0.2 ± 0.18	0.13 (0–0.25)	0.21 ± 0.2	0.13 (0.13–0.25)	0.17 ± 0.19	0.13 (0–0.25)	0.366
**Anxious cognitive**	**0.64 ± 0.34**	**0.67 (0.33–1)**	**0.59 ± 0.34**	**0.67 (0.42–0.83)**	**0.56 ± 0.35**	**0.67 (0.33–0.83)**	0.490
**Anxious somatic**	0.4 ± 0.24	0.33 (0.33–0.5)	0.37 ± 0.26	0.33 (0.17–0.58)	0.35 ± 0.28	0.33 (0.17–0.5)	0.496

HT- Hashimoto thyroiditis

* Kruskal Wallis test was used to test the difference between all groups togethe, Tukey and Mann Whitney test were used as poshoc tests for multiple comparation

When difference in depressive temperament was compared, there was significant difference between 3 groups. Post hoc test revealed that difference was significant between hypothyroid and control group, hypothyroid had a higher score for depressive temperament (p<0.001). Also, difference was significant between euthyroid and control group, euthyroid had a higher score for depressive temperament (p = 0.023). There was no significant difference between hypothyroid and euthyroid HT (0.489).

Significant difference between 3 groups was found for ciclotymic temperament (p<0.001). Post hoc test revealed that difference was significant between hypothyroid and control group, hypothyroid had a higher score for cyclothymic temperament (p<0.001). Also, difference was significant between euthyroid and control group, euthyroid had a higher score for cycolothymic temperament (p = 0.001). There was no significant difference for ciclotymic temperament between hypothyroid and euthyroid HT (0.704).

Summarizing the comparison of the groups with HT and the control group, a statistically significant difference was observed regarding the rate of severity (mild, moderate elevated or strongly elevated) degree of distress, depression, anxiety and somatization on the 4DSQ questionnaire. Levels of compared 4DSQ questionnaire were compared between three groups in Table 3. Levels of distress were higher in Hashimoto group when we compared scores on 4DSQ scale (p = 0.005). There was significant difference between distress domain, with highest level in hypothyroid HT group (p = 0.05). When levels of depression and anxiety were compared between groups, there was no siginificant difference. When levels of somatisation are compared, there was difference in somatisation scores between three groups, with highest level in hypothyroid HT group (p = 0.023).

**Table 3:** The severity of psychosomatic symptoms to the 4DSQ score in study population.

Variables 4DSQ	Hypothiroid HT group	Euthyroid HT group	Control group	P value
**Distress** (Med (25th-75th percentile))	13 (6–24)	10 (6–17)	7 (4–13)	**0.005¥**
**Depression** (Med (25th-75th percentile))	0 (0–2)	0 (0–2)	0 (0–1)	0.223¥
**Anxiety** (Med (25th-75th percentile))	4 (1–9)	1 (0–7.5)	2 (0–5)	0.223¥
**Somatisation** (Med (25th-75th percentile))	17 (11–26)	14 (8.5–21.5)	11 (6–19)	**0.023¥**

HT- Hashimoto thyroiditis

¥Kruskal Wallis test was used to test the difference between test scores

Correlation matrix between all groups of respondents with dominant affective temperament of TEMPS-A and level of psychosomatic symptoms of 4DSQ questionnaire is shown in Table 4. Higher scores for TEMPS-A domains were correlated with higher scores for all dimensions in 4DSQ in hypothyroid HT group. In euthyroid HT group there was positive significant correlation between depressive and ciclotymic temperament and distress, depression and somatisatiom for only ciclotymic temperament dimension on 4DSQ questionnaire. Cyclotimic temperament correlated postive with all 4 dimensions on 4DSQ questionnaire in control group.

**Table 4:** Correlation matrix between groups, affective temperaments and psychosomatic symptoms.

TEMPS A		Hypothiroid HT group				Euthyroid HT group				Control group			
		distress	depression	anxiety	somatisation	distress	depression	anxiety	somatisation	distress	depression	anxiety	somatisation
**Depresive**	r*	.595**	.653**	.494**	.385**	.433*	.464*	0.282	0.248	0.218	.256*	0.112	.347**
	p	<0.001	<0.001	<0.001	0.006	0.035	0.022	0.182	0.243	0.064	0.029	0.345	0.003
**Ciclotymic**	r	.666**	.692**	.473**	.319*	.637**	.549**	0.36	.574**	.655**	.632**	.533**	.518**
	p	<0.001	<0.001	0.001	0.026	0.001	0.005	0.084	0.003	<0.001	<0.001	<0.001	<0.001
**Hypertimic**	r	-.359*	-0.27	-.327*	-0.069	0.042	-0.15	-0.214	-0.152	-.355**	-.419**	-.289*	-0.23
	p	0.011	0.061	0.022	0.637	0.844	0.484	0.315	0.478	0.002	<0.001	0.013	0.051
**Irritabile**	r	.494**	.463**	.453**	.395**	0.065	-0.012	0.182	0.124	.313**	0.226	0.062	0.105
	p	<0.001	0.001	0.001	0.005	0.763	0.957	0.394	0.564	0.007	0.054	0.6	0.377
**Anxious cognitive**	r	.572**	.529**	.502**	.419**	0.049	0.04	0.093	-0.144	.355**	.322**	.414**	.344**
	p	<0.001	<0.001	<0.001	0.003	0.82	0.854	0.664	0.504	0.002	0.006	<0.001	0.003
**Anxious somatic**	r	.440**	.328*	.315*	.581**	-0.017	0.12	-0.343	-0.037	.465**	.331**	.259*	.265*
	p	0.002	0.022	0.028	<0.001	0.936	0.577	0.101	0.865	<0.001	0.004	0.027	0.023

*r Spearman correlation coeficient; *p<0.05 **p<0.001

## Discussion

To the best of our knowledge, this is the first study to apply the TEMPS-A questionnaire for evaluating the AT in women with HT. In our study, we examined the dominant style of AT and its correlation with the levels of psychosomatic symptoms in women with HT compared to control, clinically healthy women without HT. The study provides significant data on relation between AT and psychosomatic symptoms in women with hypothyroid HT and euthyroid HT, i.e. the difference between them, and in comparison with the control group.

In our study, we found no significant differences in Body Mass Index (BMI), marital and work status, education, and employment between the grups with HT and control group. The only notable difference between these groups was in laboratory parameters, which was in accordance with the criteria for inclusion in the study (Table 1). First, we examined the representation of affective temperament in our examined groups, shown in Table 2.

Our results, as measured by TEMPS-A, indicated that hyperthymic AT was prevalent across all groups studied, with the lowest score observed on the depressive AT subscale (Table 2). The dominance of hyperthymic AT has been confirmed in several studies on AT [23, 25–28]. The highest score for hyperthymic AT was opserved in the control group, however, no statistically significant difference was found between the studied groups in this style of AT. Additionally, our study observed higher scores for anxiety cognitive and cyclothymic AT types among the group with hypothyroid HT, which is consistent with previous findings in other autoimmune diseases [21]. In the groups with HT, hypothyroid and euthyroid have a high score on the TEMPS-A scale, in addition to hyperthymic, they also have anxious congitive and cyclothymic AT, which has a low score in our healthy subjects.

Our study found a statistically significant difference between the groups of subjects with HT and the control group of healthy subjects, in the presence of the depressive and cyclothymic AT (Table 2). Cyclothymic AT had the higher score in groups with HT. This results suggests that women with HT may be more susceptible to mood disorder and characterized by unstable and rapidly changing mood, fluctuations in energy and feelings, as compared to healthy subjects matched for gender and age. According to the previous research, our subjects with newly discovered HT with a high score for cyclothymic AT can be described as persons who experience intense feelings, are more prone to emotional outbursts, are impulsive and unstable, but also have mood swings from high to low, i.e. they are between polar opposite emotional states, between depression and hyperthymia [16, 26, 27].

In addition, in subjects with HT, a higher score on anxious cognitive AT subscale was also present (Table 2). Our results, like results from numerous other studies, suggest that women with newly diagnosed HT can be described as individuals prone to worries and overthinking, who often experience physical and mental tension, incapable of relaxation, prone to negative experiences [26, 27].

Our results indicate that there are no significant differences between women with hypothyroid HT and euthyroid HT according to dominant styles of AT as well as psychosomatic symptoms (Table 3). In our examined groups with HT, there are higher levels of psychosomatic symptoms compared to the control group, as measured by the 4DSQ scale. In groups of subjects suffering from HT, there were moderate elevated distress and somatization, according to 4DSQ. In both groups of subjects with HT, we found a statistically significant difference in level of distress in groups with HT, compared to the control group (p = 0. 005) (Table 3). The level of distress in the group of subjects with hypothyroid HT is more pronounced compared to the other two groups of subjects. Such results confirmed the results of the previous studies, indicating that patients with HT are additionally overwhelmed with distress [12, 30, 31]. Long-term expressed stress can change the course of the disease, i.e. lead to the worsening of other symptoms associated with it, depression, anxiety and somatization, so it is necessary to take into account the level of these symptoms at the beginning of the disease so that women with newly discovered HT can be holistically monitored in their further treatment.

In both groups of patients with HT, hypothyroid and euthyroid, we did not find high levels of depression and anxiety, according to 4DSQ questionaire, in contrast to previous studies showing that HT presented a risk of developing depression and anxiety [9–12, 30–32]. Our results are contradictory to the studies showing that the subjects with HT have a greater risk of developing anxiety, compared to the group of healthy subjects [5, 9, 31] and possible connecion between depression and chronic autoimmune thyroiditis [5, 33]. Our results are similar to those of other studies, in a large population study no statistically significant association between thyroid gland dysfunction and the presence of depressive and anxiety disorders was found [8]. Our results are similar to results from other studes that claim that elevated TPO antibody levels are not indicators of depression [34, 35]. Although our subjects do not differ significantly in terms of higher AT scores according to the TEMPS-A scale, they differ significantly in the level of distress and somatization. According to our results, there is statistically significant difference was in the level of expressed somatic symptoms between the groups with HT and control, healthy subjects (p = 0.023) (Table 3). We found that at the beginning of the disease, women with HT have a very pronounced somatization associated with distress which is also stated in other research [25]. A higher level of distress in women with HT is considered a psychological mechanism of the disease, but also an additional psychological burden in these subjects. In our subjects with hypothyroid and euthyroid HT, there is no difference in the manifestation of psychosomatic symptoms. We proved that the burden of psychosomatic symptoms in our subjects is independent of thyroid gland dysfunction, but that the level of psychosomatic symptoms differs in relation to healthy subjects in distress and somatization. These results are similar to the results of other studies, where it is stated that psychosomatic symptoms may be a consequence of stress [30, 36], and that the presence of elevated TPO antibodies is associated with worse physical and mental health [37]. Our results indicates that it is necessary to holistically observe patients with HT and identify the somatic conditions they may face in the beginning of their illness. In further studies, it is necessary to pay more attention to the experience of unpleasant physical symptoms in patients with HT.

Our results showed a significant positive correlation between all types of AT in patients with hypothyroid HT and levels of psychosomatic symptoms, with the except for hyperthymic AT. This AT which has a significant, moderately negative correlation with with symptoms of distress and anxiety (Table 4). Previous research supports the finding that hyperthymic AT may help prevent negative emotional reactions [28, 29, 38]. Our study indicates that hyperthymic AT may play a protective role in the expression of distress and anxiety in women with hypothyroid HT, as we see in other research [18]. In contrast, there was no connection found between hyperthymic AT and depression and somatization, suggesting a lack of influence of this temperament on existing higher level of pronounced somatization in our subjects. These results support the results of earlier research that state that people with hyperthymic AT can better cope with somatic problems [39].

We assume that higher scores on the TEMPS-A scale for depressive, cyclotymic, irritable, anxious cognitive and anxious somatic AT represent a higher risk for the occurrence of a higher level of psychosomatic symptoms, according to the 4DSQ questionnaire in women with hypothyroid HT (Table 4). Our findings align with existing literature and suggest that the dominant affective temperament can serve as a manifestation of mood disorders [28, 29, 40, 41] while simultaneously can affect somatic disorders [29, 38, 39]. It is necessary to pay attention to newly diagnosed patients with hypothyroid HT, because dominant AT can affect the experience of stress and its consequences, it can determine the level of psychosomatic symptoms [25, 32, 40].

According to our results, cyclothymic AT, which is statistically significantly different between our groups of respondents (Table 2), is associated with almost all psychosomatic symptoms in both groups of subjects (Table 4). There was no association between AT and the level of anxiety in subjects suffering from euthyroid HT, hence we can confirm that they have less risk of developing anxiety symptoms, relative to different types of AT. In subjects with euthyroid HT, there was a moderate positive correlation between depressive and cyclothymic AT, with distress and depression, as well as with somatic symptoms in cyclothymic AT. According to Walsh and colleagues [28], people with this AT show negative emotions, irritability and anxiety, and experience more distress, which we can see in our subjects and in our subjects with euthyroid HT according to the 4DSQ scale. Our test subjects with euthyroid HT with higher scores for cyclothymic AT may feel more distress, depression and somatization, which further increase the existing symptoms of the disease and thereby reduce her capacity to successfully adapt to her disease in everyday life situations. These results support the results of other research, which state that the dominance of cyclothymic AT, had a great possibility to develop somatic symptoms, anxiety and depressive disorders can occur [16, 17, 25–27, 29]. According to our results, there is no significant correlation between hyperthymic, irritable, anxious cognitive and anxious somatic affective temperaments with the severity of the development of psychosomatic symptoms in subjects with euthyroid HT. We did not find that high scores on these subscales of Akiskal’s temperaments influence an increased level of psychosomatic symptoms, so we can indicate that the increased level of distress and somatization in these subjects is a consequence of the existence of HT disease. In our study, in groups with hypothyroid and euthyroid HT, there was an association between a very represented cyclothymic AT type, and higher levels of expressed distress. Previous studies have also reported that individuals with this AT exhibit heightened reactivity to stress [16, 18, 28].

Also, as with the groups with HT, the group of control, healthy subjects differs from those with HT in the association of AT with psychosomatic symptoms. When we look at our results when examining the connection between AT and psychosomatic symptoms in our healthy subjects, we can confirm that high scores for cyclothymic and anxious-cognitive AT can be a risk for the development of all psychosomatic symptoms in these subjects. In addition to the high scores for these AT in healthy subjects, the highest score for hyperthymic AT is the highest, and according to our results, it has a significant negative association with stress, depression and anxiety, while the negative association with somatization was close to the consequent level. These findings point to the conclusion that different styles of AT are differently associated with psychosomatic symptoms, i.e. they differ in all examined groups. We could say that cyclothymic AT have the highest correlation in patients with HT and healthy subjects. Women with hypothyroid HT, who also have high score for cyclothymic AT have protection against the appearance of distress, anxiety and depression. Our results are consistent with previous studies showing that this type of AT is likely to have an impact on somatic conditions even in other diseases [29, 38].

We found that there was a difference between groups with HT and the association of dominant AT with psychosomatic symptoms. This kind of data was not expected, but it indicates that subjects with euthyroid HT, according to AT measured using a TEMPS-A scale, may be less susceptible to psychosomatic symptoms compared to subjects who also suffer from HT, but with reduced thyroid function. However, we did not notice any discrepancies between these two groups regarding the type of affective temperament (Table 2) and psychosomatic symptoms (Table 3), which can be attributed to the small number of subjects with euthyroid HT who participated in our study. A significant association of dominant hyperthymic AT with psychosomatic symptoms was not found in the group of subjects with euthyroid HT. So far, no studies have been conducted comparing and describing AT and psychosomatic symptoms in separate groups of subjects with euthyroid and hypothyroid HT. Additional prospective research is needed to compare these two groups of subjects with HT. Future research should be focused on more detailed monitoring of the outcome of the connection between AT and the level of psychosomatic symptoms in patients with HT.

## Conclusions

This study highlights the association between AT and HT in women with HT at the beginning of the disease. We found that higher psychosomatic symptoms as aggravating factors of this disease are associated with AT features. The findings of this study suggest a significant association between dominant affective temperaments and psychosomatic symptoms in women with hypothyroid HT. The research found differences between hypothyroid/euthyroid HT groups and the control group in terms of depressive and cyclothymic AT. Differences were also found in distress and somatization levels between the HT groups and the control group. The research found differences between study groups in the association between AT and levels of psychosomatic symptoms. Further research with a larger sample size is necessary to more clearly define the associations between affective temperaments and psychosomatic symptoms in women with euthyroid HT and hypothyroid HT.

### Limitations

The case-control type of the study, which does not allow the generalization of the findings and relatively small sample of patients for better assessment of the affective temperament. We do not have data on the number of HT patients in our country. Because this is the first study concerning AT in women with HT, it was impossible to calculate the appropriate sample size for different groups of patients suffering from HT and for healthy subjects. This research did not take differences in relation to gender because during our research we came across a very small number of male subjects, which is in accordance with the literature that HT is more common in women in the ratio of 1:10 [3].

## Supporting information

S1 File Data base of affective temperament and psychosomatic symptoms in study groups. (XLSX)

S1 Table Demographic and clinical data of study population. (XLSX)

S2 Table Affective temperament by TEMPS-A in study population. (XLSX)

S3 Table Psychosomatic symptoms in study population. (XLSX)

S4 Table Correlation matrix between groups, affective temperaments and psychosomatic symptoms. (XLSX)
